# The Impact of High-Fructose Diet and Co-Sensitization to House Dust Mites and Ragweed Pollen on the Modulation of Airway Reactivity and Serum Biomarkers in Rats

**DOI:** 10.3390/ijms25168868

**Published:** 2024-08-15

**Authors:** Răzvan-Ionuț Zimbru, Elena-Larisa Zimbru, Valentin-Laurențiu Ordodi, Florina-Maria Bojin, Daniela Crîsnic, Manuela Grijincu, Silvia-Nicoleta Mirica, Gabriela Tănasie, Marius Georgescu, Ioan Huțu, Laura Haidar, Virgil Păunescu, Carmen Panaitescu

**Affiliations:** 1 Center of Immuno-Physiology and Biotechnologies, Department of Functional Sciences, “Victor Babes” University of Medicine and Pharmacy, 300041 Timisoara, Romania; razvan.zimbru@umft.ro (R.-I.Z.); elena.zimbru@umft.ro (E.-L.Z.); valentin.ordodi@upt.ro (V.-L.O.); florinabojin@umft.ro (F.-M.B.); crisnic.daniela@umft.ro (D.C.); gtanasie@umft.ro (G.T.); georgescu.marius@umft.ro (M.G.); vpaunescu@umft.ro (V.P.); cbunu@umft.ro (C.P.); 2 OncoGen Center, Pius Brinzeu County Clinical Emergency Hospital, 300723 Timisoara, Romania; 3 Chemistry and Engineering of Organic and Natural Compounds Department, University Politehnica Timisoara, 300006 Timisoara, Romania; 4 Faculty of Sport and Physical Education, West University of Timisoara, 300223 Timisoara, Romania; nicoleta.mirica@e-uvt.ro; 5 Horia Cernescu Research Unit, Faculty of Veterinary Medicine, University of Life Sciences “King Michael I of Romania”, 300645 Timișoara, Romania; ioan.hutu@fmvt.ro

**Keywords:** allergy, ragweed pollen, house dust mites, asthma, obesity, inflammation

## Abstract

The topic of ragweed pollen (RW) versus house dust mites (HDMs) has often been deliberated, but the increasing incidence of co-sensitization between them has been scarcely addressed. Utilizing *Sprague Dawley* rats, we explored the effects of co-sensitization with the combination of HDMs and RW pollen extracts in correlation with high-fructose diet (HFrD) by in vitro tracheal reactivity analysis in isolated organ bath and biological explorations. Our findings unveiled interrelated connections between allergic asthma, dyslipidemia, and HFrD-induced obesity, shedding light on their compounding role through inflammation. The increased CRP values and airway hyperresponsiveness to the methacholine challenge suggest a synergistic effect of obesity on amplifying the existing inflammation induced by asthma. One of the major outcomes is that the co-sensitization to HDMs and RW pollen led to the development of a severe allergic asthma phenotype in rats, especially in those with HFrD. Therefore, the co-sensitization to these allergens as well as the HFrD may play a crucial role in the modulation of systemic inflammation, obesity, and airway reactivity.

## 1. Introduction

Asthma is a complex respiratory pathology characterized by heterogeneous features, the most significant being the airway inflammation and hyperresponsiveness [1]. The allergic asthma, as a clinically relevant phenotype, can be induced in animal models by repeated contact with the airborne antigens, triggering the allergic reaction which would sustain chronic inflammation and develop progressive airway remodeling [2,3].

House dust mites (HDMs) and ragweed (RW) pollen are among the most common inducers of respiratory allergies, having an important impact on the quality of life of the sensitized patients [4].

The current research represents the first comprehensive investigation into the impact of co-sensitization to HDMs and RW pollen extracts in rats and examines the main differences between the asthma endotypes induced by monosensitization and polysensitization to these allergens. Furthermore, the complementary influence of obesity induced by a high-fructose diet (HFrD) in the allergic diseases can illustrate the intricacy of these conditions in rats.

The Global Initiative for Asthma (GINA), a medical guidelines organization, recognizes asthma coexisting with obesity as the most prevalent clinical phenotype of the disease [1,5]. It is noteworthy that nearly 60% of individuals with severe or refractory asthma also exhibit obesity. Furthermore, overweight or obese status is linked to a 1.5- to 2.5-fold elevation in the likelihood of developing asthma [6].

To understand the intricate relationship between asthma and obesity, it is crucial to acknowledge that adipose tissue functions as a metabolically active organ, producing various mediators that sustain chronic and low-grade systemic inflammation. It has been proposed that this inflammatory condition could modulate airway inflammation and potentially contribute to the onset of asthma in obese individuals [7]. Consequently, adipocytes secrete a range of molecules known as adipokines, of primary significance being leptin, resistin, chemerin, interleukin 6 (IL-6), C-reactive protein (CRP), tumor necrosis factor alpha (TNF-α), apelin, and visfatin [7,8,9]. Acting as a pleiotropic cytokine, IL-6 is believed to significantly influence the pathogenesis of both asthma and obesity [6].

Epidemiological research has identified a potential link between obesity and allergic asthma, suggesting that obesity serves as both a predisposing factor for asthma development and may exacerbate its severity; however, some findings have been inconclusive [10,11,12]. A causal relationship has been observed between abdominal obesity and asthma severity [12,13,14]. Numerous research findings suggest that obesity, particularly abdominal obesity, heightens the likelihood of developing asthma and worsening asthma symptoms, including those triggered by allergens [12,14,15].

The high-fructose diet could have an impact on asthma by contributing to the development of dyslipidemia and obesity as well as through various other mechanisms [16]. Based on the “fructose hypothesis”, inadequate absorption of fructose in the intestine leads to the production of proinflammatory advanced glycation end products due to interactions between unabsorbed fructose and incompletely digested dietary proteins. Upon absorption, these products may contribute to the development of asthma [17,18]. Fructose malabsorption typically arises following the consumption of foods and drinks with a high ratio of fructose to glucose, but not after consuming sucrose or equivalent amounts of fructose and glucose separately [19].

To investigate the dietary impact on allergic asthma, studies have explored the pulmonary response following HDM-challenge, comparing the animals with ad libitum access to food with those under dietary restriction. Results indicate that rats with unrestricted food access exhibit enhanced immune function and inflammatory response, which is notably reduced in rats under dietary restriction, therefore emphasizing the influence of diet on the allergic reaction [20].

Several researchers have explored the relationship between asthma and blood lipid profile, with conflicting results [16,21]. One of the aims of this study is to investigate, using animal models, the intricate relationship between allergic asthma and HFrD-induced dyslipidemia and obesity.

CRP, an inflammatory marker, is a significant inflammation-sensitive plasma protein produced by the liver. Its levels rise in response to inflammation, often paralleling IL-6 secretion by macrophages and T cells, up to 1000-fold within just 24 h of inflammation onset [22]. Consistent with allergic disease models, the imbalance between Th1 and Th2 responses are recognized in allergic inflammation. Upon exposure to the allergens, the Th2 response characteristically involves an increase in interleukin (IL)-4, IL-5, and IL-13 [23], while IgE-independent mechanisms of allergic inflammation may be explained by the involvement of the innate immune system [23,24,25].

Increased IgE levels were noted in asthmatic rats challenged with ovalbumin (OVA) and were correlated with airway hyperresponsiveness and other asthma features, including bronchoconstriction, heightened vascular permeability, and enhanced mucus production. Elevated IgE levels in allergic asthma are recognized as a key predisposing factor in the onset of the disease [22,26].

The asthma features were developed by sensitizing the rats to OVA or to the following allergen extracts: HDMs, RW pollen, or the combination of HDMs and RW pollen.

There were three main methods employed to assess the changes induced by asthma and HFrD in rats. Metabolic parameters were evaluated through serum biomarkers, including lipid profile and plasmatic inflammation markers. The isolated organ bath served as a valuable experimental approach for investigating the effects of allergen challenge on airway hyperresponsiveness, utilizing methacholine chloride as a bronchoconstrictor agent on isolated tracheas. Providing a controlled and reproducible in vitro environment, this physiological exploration focuses on assessing the contractile force or tension changes in the airways, contributing to understanding the physiopathological changes and establishing the dose–response relationships to agonists. Histological examination is a central method for identifying the characteristic features of asthma in vitro, including lung inflammation, luminal diameter, and wall thickness in the airways, which indicate the extent of allergen-induced affliction in allergic asthma. This examination is essential for confirming the diagnosis of asthma in rats, providing detailed insights into the pathological changes occurring in the airways, thereby supporting the accuracy of the experimental model [27].

In the current research, we conclusively establish that co-sensitization to HDMs and RW pollen has a significant impact on airway hyperresponsiveness compared to monosensitization to either HDMs, RW pollen, or OVA. This effect was substantially aggravated by the HFrD and could indicate a higher disease severity even shortly after its onset.

## 2. Results

### 2.1. The Impact of Dietary Factors and Allergic Responses on Serum Biomarkers

#### 2.1.1. Measurement of Anthropometric Parameters

The initial body weight (BW) exhibited no statistically significant differences across experimental groups (311.33 g ± 15.26 g, *p* > 0.05). BW evolution is presented in Table 1.

Rats with HFrD over 12 weeks showed a notable increase in body weight compared to the standard diet subjects (383.93 g ± 24.72 g vs. 326.83 g ± 19.23 g, *p* < 0.05). In contrast, the experimental groups with allergic asthma induced by OVA, HDMs, RW, or the combination of the last two allergens displayed a plateau in BW gain after the initial allergen challenge until sacrifice, in comparison to the control group.

**Figure 1:**
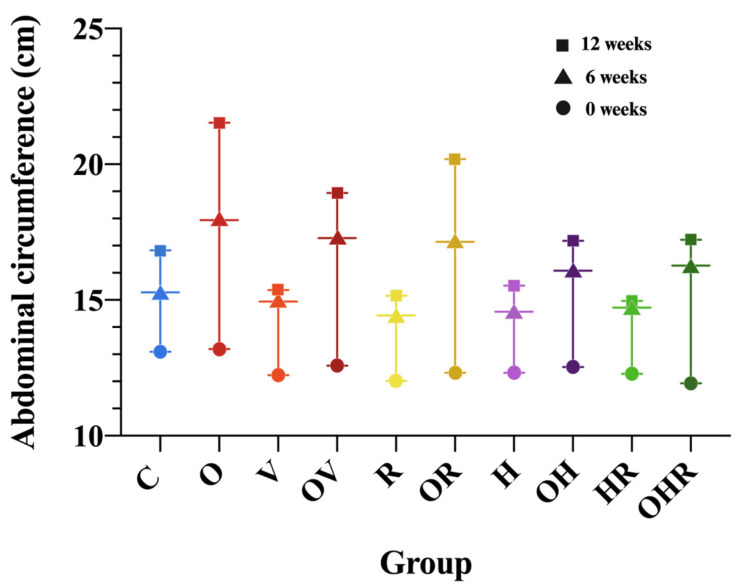
Abdominal circumference evolution at 0, 6, and 12 weeks from the initiation of group-specific conditions. The groups are depicted as follows: C = control; O = high-fructose diet; V = ovalbumin sensitization; OV = high-fructose diet and ovalbumin sensitization; R = ragweed pollen sensitization; OR = high-fructose diet and RW sensitization; H = house dust mite sensitization; OH = high-fructose diet and HDM sensitization; HR = HDM and RW sensitization; OHR = high-fructose diet and HDM and RW sensitization.

Rats in the HFrD groups exhibited a significant increase in abdominal circumference (AC) compared to the control group (an increase of 4.41 ± 0.82 cm at 6 weeks and 6.82 ± 1.68 cm at 12 weeks, *p* < 0.0001). Similar to the progression of BW, the AC in allergic asthma groups showed a plateau in the rate of increase following the initial allergen exposure, persisting until sacrifice (Figure 1).

#### 2.1.2. Serum Lipidic and Glucose Profiles in Sensitized Rats

**Figure 2:**
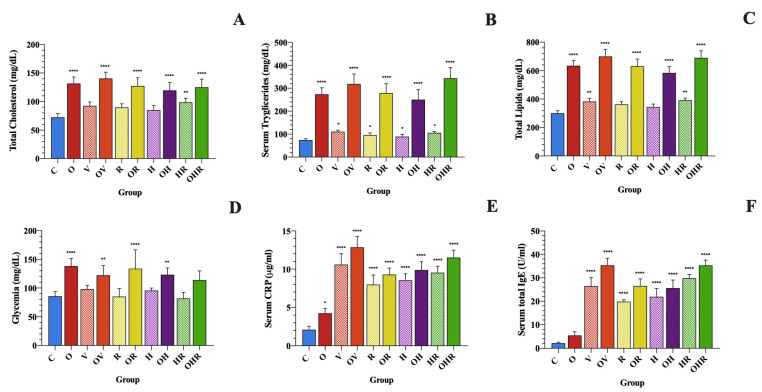
Blood analysis parameters of rats after a 12-week regimen. (**A**) Serum total cholesterol. (**B**) Serum tryglicerides. (**C**) Serum total lipids. (**D**) Glycemia. (**E**) Serum CRP. (**F**) Serum total IgE. Data are shown as mean ± SD (n = 6 in each group). IgE = Immunoglobulin E, CRP = C-reactive protein. The groups are depicted as follows: C = control; O = high-fructose diet; V = ovalbumin sensitization; OV = high-fructose diet and ovalbumin sensitization; R = ragweed pollen sensitization; OR = high-fructose diet and RW sensitization; H = house dust mite sensitization; OH = high-fructose diet and HDM sensitization; HR = HDM and RW sensitization; OHR = high-fructose diet and HDM and RW sensitization. Statistical analysis was performed using one-way analysis of variance (ANOVA) followed by Tukey’s multiple comparison test. * *p* < 0.05, ** *p* < 0.01 and **** *p* < 0.0001 vs. control group.

At the beginning of the study, there were no statistically significant differences among the groups regarding lipid and glucose profiles (Figure 2A–D). The biochemical parameters identified after the 12-week regimen are shown in Figure 2. HFrD administration in rats led to increased body weight and abdominal circumference, triglycerides levels, glycemic values, and elevated CRP levels (Figure 2A,D,E). Additionally, allergic asthma appears to exacerbate these parameters, further increasing the risk and worsening the dyslipidemia and obesity.

The lipidic profiles of total cholesterol (TC), triglycerides (TG), and total lipids (TL) in the HFrD groups (OV, OR, OH, and OHR) were significantly higher than those found in controls (*p* < 0.0001) (Figure 2A–C). Also, the co-sensitization with both RW and HDMs significantly increased the TC by 36.1%, TG by 42%, and TL by 30.3% (*p* < 0.001). In allergic asthma groups (V, H, R, HR), significantly higher triglyceride values were observed compared to controls (*p* < 0.01). Moreover, we observed that co-sensitization to both HDMs and RW has a greater impact on triglyceride levels compared to HDMs alone (*p* < 0.01).

Furthermore, rats that consumed water fortified with 40% fructose for 12 weeks showed significantly higher glycemic values (126.10 ± 20.38 mg/dL, *p* < 0.0001) compared to those on a standard diet (89.21 ± 10.85 mg/dL) (Figure 2D).

#### 2.1.3. Modulation of Serum C-Reactive Protein and Serum Total IgE Antibody Levels Following Allergen Sensitization

The potential influences of a multifactorial regimen, consisting of a combination of previously mentioned allergen sensitizations and diet, were investigated through a series of serum parameters.

The current results suggest that sensitization with HDMs, RW, or both significantly elevates CRP compared to the control group (*p* < 0.001) (Figure 2E). Therefore, CRP could be a valuable indicator for detecting systemic inflammation in allergic asthma. Compared to the standard diet groups that had the same sensitization protocol, rats that received HFrD had 16.5% (OR vs. R), 15.5% (OH vs. H), and 20.8% (OHR vs. HR, *p* < 0.05) higher serum CRP levels than the corresponding standard diet group, although the differences were statistically significant only in the co-sensitization groups. The serum CRP levels in the groups challenged with HDMs, RW, or both were similarly elevated compared to the OVA-challenged group and were significantly increased compared to control rats (*p* < 0.0001).

**Figure 3:**
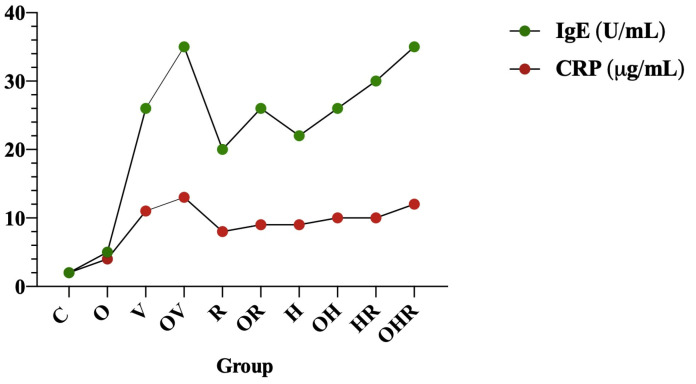
Correlation between serum CRP and IgE levels across the experimental groups. The groups are depicted as follows: C = control; O = high-fructose diet; V = ovalbumin sensitization; OV = high-fructose diet and ovalbumin sensitization; R = ragweed pollen sensitization; OR = high-fructose diet and RW sensitization; H = house dust mite sensitization; OH = high-fructose diet and HDM sensitization; HR = HDM and RW sensitization; OHR = high-fructose diet and HDM and RW sensitization.

Allergen challenge significantly increased IgE serum levels to a 13.6-fold increase in the V group, a 11.4-fold increase in the H group, a 10.4-fold increase in the R group, and a 15.2-fold increase in HR group in comparison with the control group (Figure 2F). Alternatively, in contrast to the standard diet groups following the same sensitization protocol, obese rats exhibited significantly higher total serum IgE levels (*p* < 0.05 in OV vs. V, OH vs. H, and OHR vs. HR groups). Figure 3 illustrates the correlation between systemic inflammation, as measured by serum CRP levels and total serum IgE levels (r = 0.979).

### 2.2. Revealing the Impact of High-Fructose Diet and Co-Sensitization in Contrast to Monosensitization to Allergenic Extracts in Rats

**Figure 4:**
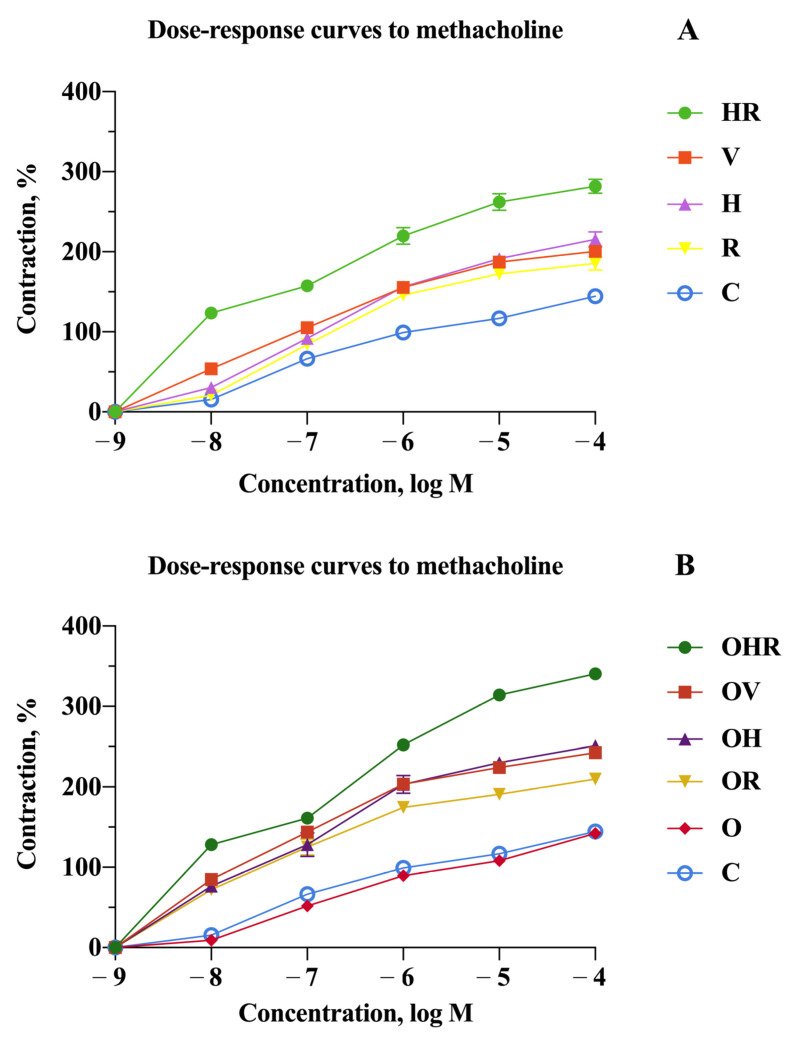
Dose–response curves to methacholine in the organ bath system for the groups without high-fructose diet (**A**) and for the groups with high-fructose diet (**B**). Results from each point are expressed as the mean percentage ± SD of the contraction value normalized to the tension developed by 10^−6^ M methacholine concentration in the control group. Each value is derived from two experiments per rat, with a total of six rats per group contributing to the dataset. The groups are depicted as follows: C = control; O = high-fructose diet; V = ovalbumin sensitization; OV = high-fructose diet and ovalbumin sensitization; R = ragweed pollen sensitization; OR = high-fructose diet and RW sensitization; H = house dust mite sensitization; OH = high-fructose diet and HDM sensitization; HR = HDM and RW sensitization; OHR = high-fructose diet and HDM and RW sensitization.

To evaluate the changes in airway responsiveness in the sensitized animals, tracheas were suspended in the organ bath system, where the contractions to mediators were induced. Methacholine chloride, a nonselective muscarinic receptor agonist, acts on Gq–coupled M3 cholinergic receptors in the airways. This interaction mediates the intracellular calcium influx into smooth muscle cells, inducing bronchoconstriction. The titrated concentrations of this mediator ranged from 10^−8^ to 10^−4^ M, and the corresponding dose–response curves were measured and are depicted in Figure 4.

Figure 4 shows the relationships between dose–response curves to methacholine for all the studied groups. One prominent aspect is that rats with HFrD consistently exhibit greater contraction tension in response to methacholine across all concentrations compared to rats without HFrD but sensitized to the same allergen (*p* < 0.0001 in OV vs. V, OR vs. R, OH vs. H, and OHR vs. HR groups). Another important characteristic is that the stimulation threshold decreases. While the response to methacholine at dilutions of 10^−8^ M and 10^−7^ M may not be substantial for some sensitized animals, the groups that had the high fructose-diet combined with the allergenic sensitization present a significant response even at these higher dilutions (*p* < 0.0001 in C vs. OV, OH, OR, and OHR groups).

The strongest contraction was induced by the combination of HDMs and RW pollen. Tracheal reactivity was significantly more pronounced across all methacholine concentrations in the OHR group compared to the HR group (*p* < 0.0001). While no stronger response than the classic sensitization to ovalbumin was observed in cases of monosensitization to allergens, bisensitization to HDMs and RW pollen led to a significantly greater contraction for both dietary types compared to all other groups at each methacholine concentration tested (*p* < 0.0001).

Even though at high concentrations of methacholine (10^−4^ and 10^−5^ M) the response is more prominent for HDMs compared to OVA (*p* < 0.01), there is no statistically significant difference in overall reactivity induced by sensitization to these allergens when analyzing the OV vs. OH and V vs. H groups.

Tracheal reactivity in RW sensitized rats exhibited lower contractions compared to those induced by HDMs and OVA, indicating a statistically significant difference (*p* < 0.001) when comparing the groups individually, with or without HFrD.

While slightly inferior values were recorded in the group with only HFrD (O group) compared to the control group (C), there were no statistically significant differences in tracheal contractions induced by methacholine.

### 2.3. Morphologic Analysis and Characterization of Inflammatory Cell Infiltration, Bronchial Smooth Muscle Hypertrophy, and Bronchial Wall Thickness

Examination of H&E-stained tracheal and lung tissues revealed the characteristic microscopic features of asthma in all sensitized groups. Cross-sectional analysis of the bronchopulmonary system was conducted to assess inflammation, total wall thickness (WT, μm), and smooth muscle thickness (SMT, μm) of the segmental bronchi. The mean WT and SMT of segmental bronchi varied significantly among the rat groups under investigation.

**Figure 5:**
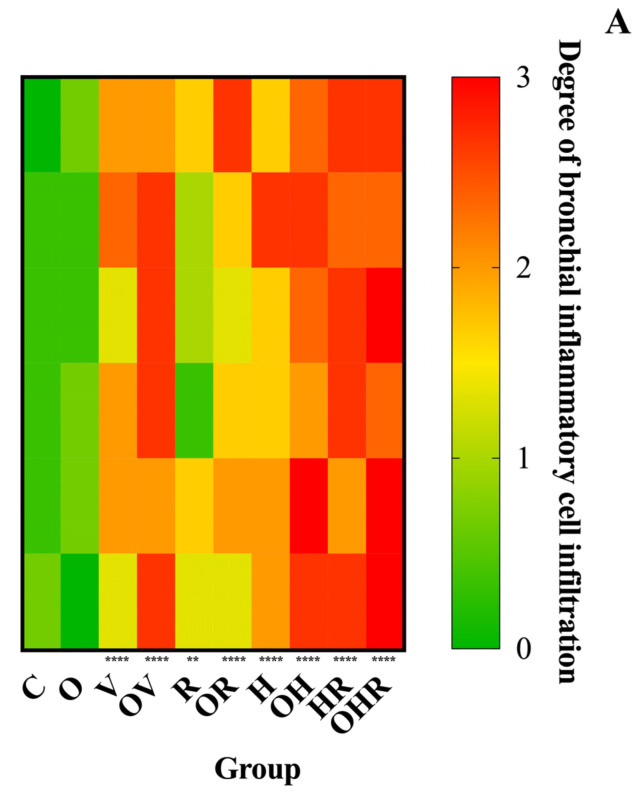

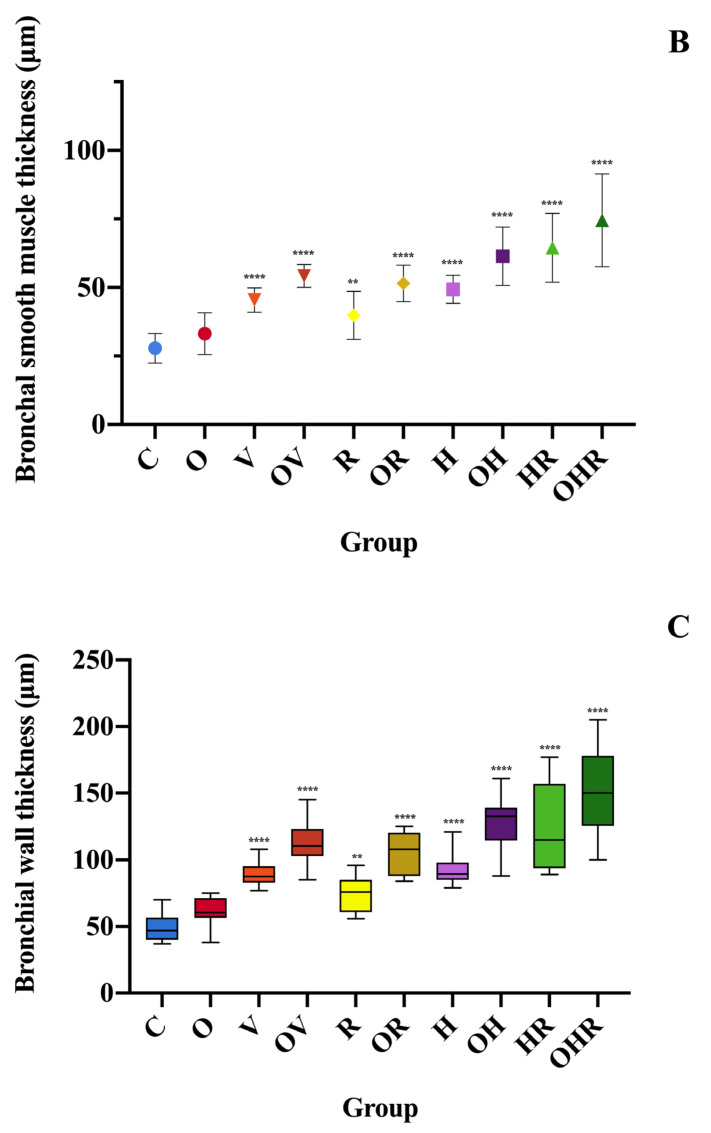
Morphological changes in the bronchial wall. (**A**) Inflammatory cell infiltration. Inflammatory cell infiltration in the bronchial wall was graded as follows: 0—no inflammation, 1—reduced inflammation, 2—moderate inflammation, 3—extensive inflammation. Data are presented as medians for each rat (n = 6 rats per group, 3 values per rat). (**B**) Segmental bronchial smooth muscle thickness. (**C**) Total segmental bronchial wall thickness. For panels B and C, data are shown as mean ± SD (n = 6 rats in each group, 3 values per rat). The groups are defined as follows: C = control; O = high-fructose diet; V = ovalbumin sensitization; OV = high-fructose diet and ovalbumin sensitization; R = ragweed pollen sensitization; OR = high-fructose diet and RW sensitization; H = house dust mite sensitization; OH = high-fructose diet and HDM sensitization; HR = HDM and RW sensitization; OHR = high-fructose diet and HDM and RW sensitization. Statistical analysis was performed using one-way analysis of variance (ANOVA) followed by Tukey’s multiple comparison test. Significance levels are indicated as ** *p* < 0.01 and **** *p* < 0.0001 compared to the control group.

Histological examination of tracheobronchial inflammation in H&E-stained lung sections was performed to assess the degree of inflammatory cell infiltration using the specified grading scale. Prominent infiltration of inflammatory cells, primarily lymphocytes, mast cells, and eosinophils, was observed in the peribronchial regions of histological sections. These pathological alterations were more pronounced in asthmatic rats from the OHR and HR groups, while the OH, H, OV, and V groups exhibited a similar pattern of infiltration (Figure 5A).

Structural modifications in bronchial smooth muscle thickness were examined between the nonasthmatic groups (C = 27.8 ± 5.3 μm; O = 33.2 ± 7.5 μm, *p* > 0.05 in O vs. C) and those challenged with allergens (V = 45.4 ± 4.3, *p* < 0.001; R = 39.8 ± 8.7 μm, *p* < 0.01; H = 49.3 ± 5.1 μm, *p* < 0.001; HR = 64.5 ± 12.5 μm, *p* < 0.001; OV = 54.2 ± 4.1 μm, *p* < 0.001; OR = 51.5 ± 6.6 μm, *p* < 0.001; OH = 61.4 ± 10.6 μm, *p* < 0.001; OHR = 74.5 ± 16.8 μm, *p* < 0.001). A marked increase in smooth muscle thickness was detected in all sensitized groups (Figure 5B).

Comparison of bronchial wall thickness between nonasthmatic groups (C = 49.8 ± 10.9 μm; O = 61.5 ± 10.4 μm, *p* > 0.05 in O vs. C) and allergen-challenged ones (V = 89.8 ± 8.9, *p* < 0.0001; R = 75 ± 11.3 μm, *p* < 0.01; H = 93.4 ± 12.9 μm, *p* < 0.0001; HR = 124.4 ± 32.3 μm, *p* < 0.0001; OV = 113 ± 16.7 μm, *p* < 0.0001; OR = 103.6 ± 18.2 μm, *p* < 0.0001; OH = 126.4 ± 22.5 μm, *p* < 0.0001; OHR = 149.4 ± 33.2 μm, *p* < 0.0001) reveals a significant increase in wall thickness in all allergic subjects (Figure 5C). Notably, the HFrD considerably impacts the allergic response, particularly seen in the augmentation of bronchial wall thickness.

The OHR group exhibited the most remarkable structural changes in the airways. Compared to the OHR group, substantial alterations in smooth muscle and wall thicknesses were also noted in the HR (*p* < 0.05), OH (*p* < 0.05), H (*p* < 0.001), OV (*p* < 0.001), and V (*p* < 0.001) groups, all of which exceeded changes seen in other groups. Extensive airway inflammation was detected, with no statistically significant differences among the OHR, HR, OH, and OV groups.

Another hallmark of asthma observed in the asthmatic groups was goblet cell hyperplasia. This effect was less pronounced in the R group and absent in the C and O groups.

## 3. Discussion

To our knowledge, this is the first study to investigate the joint effect of allergic asthma induced by HDMs and RW co-sensitization and HFrD on the airway hyperresponsiveness to methacholine and lipid profile.

The utilization of experimental animal models has been pivotal in unraveling the mechanisms underlying asthma pathogenesis and quantifying the effects of diverse allergens implicated in its development. Additionally, the response of the animal model must accurately reflect the morphological and pathophysiological changes that occur in human allergic asthma induced by the studied allergens [28].

Our findings reveal that a 40% fructose intake in drinking water leads to caloric excess, resulting in an obese state characterized by dyslipidemia and increased body weight [29,30,31,32,33]. Even so, there are also studies where additional fructose intake did not result in increased body weight, but it did lead to dyslipidemia; however, this observation is particularly notable in studies employing lower concentrations of fructose [34].

Regarding the evolution of AC in HFrD, with or without allergen sensitization and challenge, there is a significant reduction in AC at 12 weeks for the OV (18.95 ± 0.68 cm, *p* < 0.0001), OH (17.18 ± 0.73 cm, *p* < 0.0001), and OHR (17.62 ± 0.45 cm, *p* < 0.0001) groups compared to the HFrD-only group (O: 21.53 ± 0.66 cm). However, this reduction was not significant in the OR group (20.20 ± 1.00 cm, *p* = 0.06). These data are consistent with those identified by histopathologic examination and organ bath reactivity that indicated less severe responses in the ragweed-sensitized rats. In the BW evolution among HFrD groups, with or without allergen sensitization and challenge, a significant reduction in BW gain was observed at 12 weeks exclusively in the OHR group (BW: 363.17 ± 14.20 g, *p* = 0.0034) compared to the HFrD-only group (BW: 405.83 ± 13.96 g). This can be explained by decreased appetite due to asthma symptoms like dyspnea, coughing that can make eating and drinking feel uncomfortable or difficult, resulting in reduced food intake, and also the increased effort of breathing during asthma exacerbations that necessitates an intensification of energy consumption by the body and may lead to greater calorie expenditure, possibly balancing out caloric intake and leading to weight maintenance or even weight loss. The drop in body weight gain may be statistically significant only in the OHR group, as histopathological examination, organ bath reactivity, and serum biomarkers have identified it as exhibiting the most severe response to allergens, and the duration from allergen exposure until sacrifice is probably too short to determine more explicit changes.

In individuals with obesity and asthma, both pulmonary and adipose tissue may experience hypoxia due to compromised pulmonary function and oxygenation, while enlarged adipocytes face significant metabolic strain [35]. Furthermore, aside from directly impacting cellular metabolism, hypoxia triggers inflammation by activating the inflammatory mediator hypoxia-induced factor 1a (HIF-1a), which in turn stimulates NF-kB to promote the transcription of proinflammatory mediators like IL-1b and IL-6 [36]. Hypoxia serves as a link between asthma and obesity by impacting the polarization of macrophages. This highlights the significant role of macrophages in both obesity and asthma, including their ability to amplify inflammation under hypoxic conditions [15,37].

Fructose metabolism lacks a regulatory step, leading to the conversion of fructose into fatty acids and triglycerides. These findings are consistent with previous studies and support our data, demonstrating that ad libitum consumption of an enriched 40% fructose diet over 12 weeks resulted in elevated levels of TG, TC, TL, and glycemia compared to control rats. Overconsumption of fructose can exceed the liver’s processing capabilities, resulting in heightened production of fatty acids and triglycerides. This excess production contributes to elevated levels of triglycerides, results that are consistent with our study. Also, elevated fructose levels trigger cholesterol synthesis in the liver by enhancing the activity of enzymes responsible for cholesterol production. Prolonged intake of high-fructose diet is also linked to insulin resistance, leading to hyperglycemia, as reported by previous studies [38,39]. In their research, Vinding et al. (2016) discovered a correlation between elevated triglyceride levels and sensitization to aeroallergens [40]. Corresponding with prior studies, we noticed a positive association between dyslipidemia (elevated TC, TG levels) and allergic asthma (*p* < 0.05) [41].

The impact of obesity on allergic asthma extends beyond mechanical factors, as the adipose tissue also acts as an active endocrine regulator, influencing inflammation and metabolism. Metabolic disorders like dyslipidemia and insulin resistance have also been linked to asthma onset and exacerbation, though it was not clearly specified if this association is dependent on obesity [12,16,42]. Moreover, excessive fat accumulation reduces levels of the anti-inflammatory adipokines, adiponectin, omentin-1, and nesfatin-1, while elevating leptin, resistin, and chemerin production, which results in a proinflammatory status associated with asthma development [7,8,9]. The systemic inflammation associated with allergic asthma also affects lipid metabolism and glycemic control. Proinflammatory cytokines released during asthma exacerbations can disrupt insulin sensitivity and alter lipid metabolism, thereby contributing to dyslipidemia and hyperglycemia [6,43].

Elevated serum triglycerides were linked to asthma in patients with obesity, suggesting that elevated triglycerides could be a yet-unrecognized factor contributing to asthma development [44]. In our study, we found that the obese asthmatics exhibited the highest levels of TL, TC, and TG. This suggests that the low-grade inflammation characteristic of obesity intensifies the inflammation associated with asthma, leading to hyperlipidemia through a synergistic interaction. Possible mechanisms behind this synergy include the perpetuation of chronic inflammatory status by hypercholesterolemia in both asthma and obesity, as well as the interaction induced by dietary cholesterol between airway inflammation and systemic inflammation.

There seems to be a positive correlation between allergic asthma and obesity in terms of inflammation (*p* < 0.05). In obese asthmatic rats, the degree of airway inflammation is higher, accompanied by elevated CRP levels, indicating a synergic impact of obesity on pre-existing airway inflammation. The Pearson correlation coefficient for IgE and CRP levels across the ten experimental groups is 0.979, indicating a very strong positive correlation. This suggests that as IgE levels increase, CRP levels also tend to increase, demonstrating a significant association between these biomarkers in the context of allergic asthma and HFrD. Therefore, CRP could serve as a predictive indicator for both obesity and asthma.

Essential for investigating the impact of allergen sensitization in the rat asthma model was confirming that the regimen of allergen extract exposure led to airway hyperresponsiveness, the key feature of asthma. Therefore, the tracheobronchial tree was studied using the organ bath system, and serum showed the lipidic, inflammatory, and allergic responses, providing various approaches to emphasize the main distinguishing characteristics for each allergen sensitization.

Introducing a novel preclinical asthma model, we exposed the respiratory mucosa of rats to the combination of HDMs extract with ragweed pollen extract, resulting in the development of asthma-like pathology. The observed airway inflammatory response was attributed to the release of mast cell mediators, suggesting that HDMs and RW pollen concomitant exposure induces allergic inflammation and airway remodeling.

In the final part of the study, the allergic responses to repeated intratracheal HDMs and RW pollen extracts administrations were further examined by investigating the functional tracheal response to this allergenic challenge, conducted by measuring the tracheal responsiveness to mediators using the organ bath system. This showed that the most impactful sensitization was in the OHR and HR groups, therefore insinuating that the co-sensitization to these allergens could also imply the development of a severe allergic asthma form from the early stages of the disease.

**Figure 6:**
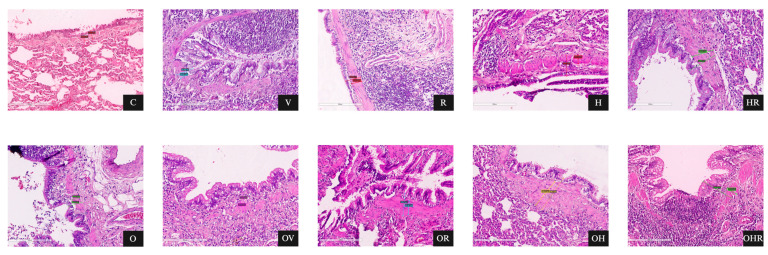
Microscopic depiction of morphological alterations in the bronchial wall in H&E-stained lung sections (20× magnification). The groups are labeled as follows: C = control; O = high-fructose diet; V = ovalbumin sensitization; OV = high-fructose diet and ovalbumin sensitization; R = ragweed pollen sensitization; OR = high-fructose diet and RW sensitization; H = house dust mite sensitization; OH = high-fructose diet and HDM sensitization; HR = HDM and RW sensitization; OHR = high-fructose diet and HDM and RW sensitization.

The OVA-induced airway inflammation model shares many similarities with the human allergic asthma. Previous studies have demonstrated that OVA exposure induces interstitial inflammation, fibrosis, emphysema, and epithelial damage in the lungs of animals, confirming sensitization [3,45]. The histopathological data obtained in this study corroborate the findings from the organ bath analysis and serum biomarkers. Similar to previous studies, inflammation scores were significantly elevated in the allergic asthma groups compared to the control group, with lung tissue exhibiting inflammation, congestion, perivasculitis, and peribronchiolitis [46]. Furthermore, the groups with HFrD associated with allergic asthma exhibited the highest inflammation scores (as shown in Figure 5A), except for the OR group. The allergic reaction induced by ragweed pollen extract had the mildest effect compared to all other sensitizations. Nevertheless, it produced statistically significant responses in several morphologic measurements, including bronchial wall thickness (R group, *p* < 0.01; OR group, *p* < 0.0001), smooth muscle thickness (R group, *p* < 0.01; OR group, *p* < 0.001), and airway inflammation (R group, *p* < 0.05; OR group, *p* < 0.001). Substantial morphological changes indicate that HDM extract sensitization in rats induced a highly potent effect, comparable to the impact of ovalbumin, which is commonly used as a standard allergen in animal studies. The association between HFrD and allergic asthma induced by both RW and HDMs seems to have the most negative impact on lung tissue histology in terms of inflammation, smooth muscle, and bronchial wall thickness (Figure 5 and Figure 6).

Exposure to allergens or environmental pollutants may trigger abnormal responses from both the innate and adaptive immune systems. Airway dendritic cells (DCs) continuously monitor inhaled air for harmful substances through cellular mechanisms along the airway mucosal barrier [47]. By employing pattern recognition receptors, such as Toll-like receptors (TLRs), to detect pathogens and to present pathogen-derived antigens to naïve T cells, these DCs act as a link between innate and adaptive immunity [48].

In the initiation of the respiratory allergic reactions, the allergen-specific CD4+ Th2 lymphocytes typically play a key role by stimulating the production of specific IgE antibodies directed at the allergens [49]. Additionally, recent research has highlighted the pivotal involvement of innate immune system activation in the pathogenesis of ragweed- and HDM-induced allergies [25,50].

The primary constituents of HDMs and RW are proteins known to elicit these adaptive Th2-skewed responses [51]. But, by serving as potent triggers for innate immune cells within the airway epithelium, they promote allergic reactions via pathways dependent on pattern recognition receptors [25].

Current findings show that the ragweed pollen acts as a functional TLR4 agonist, initiates the TLR4-dependent signaling pathways, and triggers the Th2-dominant allergic inflammation [52]. This might occur primarily through its major allergens, with Amb a 11 potentially exerting a significant impact on this signaling route [4,53]. The structural sequences alignment with other allergens revealed that Amb a 11 exhibits homology with other prominent allergens within the same protease family, including Der p 1 from house dust mites (23.5%), which might enhance a synergic allergenic effect [4]. Furthermore, Der p 1 activates dendritic cells, promoting the production of Th2 proinflammatory cytokines via a TLR4-dependent mechanism. Der p 2 may target the same process, potentially mimicking the function of MD-2 by binding to LPS and subsequently loading it, along with itself, onto TLR-4 and proposing a mechanism that could amplify its allergenicity [54,55,56]. These findings indicate that the sensitization through this pathway could play a significant role in the pathogenesis of allergic asthma.

Moreover, studies have shown that when HDMs activate Toll-like receptor 4 in the airway epithelium, it has been demonstrated to induce the secretion of innate pro-Th2 cytokines, including thymic stromal lymphopoietin (TSLP), granulocyte-macrophage colony-stimulating factor (GM-CSF), IL-25, and IL-33 [23,25,48]. These cytokines, referred to as allarmins, are key factors in the initial phase of allergic sensitization and development of asthma.

Indeed, several other Toll-like receptors may play significant roles in allergen sensitization. For instance, Der p 13, 14, and 21, based on sequence homology, could potentially facilitate allergen sensitization by activating TLR2, another type of pattern recognition receptor [25].

The TLR4 signaling pathway is recognized as a primary initiator of the inflammatory response induced by obesity. In this context, the elevation of plasma fibrinogen and CRP levels, both considered positive acute phase proteins, serves as a contributing factor in activating the TLR4 route and, consequently, exacerbating the inflammatory response [57].

Therefore, considering their similar modes of action in these allergic and inflammatory processes, it is highly plausible that a common mechanism amplifies and elicits a cumulative response in the organism. We observed that this combined response is directly proportional to the increase in factors that influence the TLR4 signaling pathways. Consequently, the combination of an HFrD and sensitization to both RW and HDMs implies a summation of similar type of stimulation, thereby resulting in a pathological response of a greater magnitude. This could provide an insightful explanation for our results, indicating an enhanced reaction to the combination of allergens and HFrD.

## 4. Materials and Methods

### 4.1. Drugs

Potassium chloride and methacholine chloride were used in the organ bath study. Ovalbumin (grade V), aluminum hydroxide (Al(OH)_3_), house dust mite extract, and ragweed pollen extract were employed in the sensitization and challenge process in order to induce and sustain allergic asthma in rats. Methacholine chloride, potassium chloride, aluminum hydroxide, and D-(−)-fructose (>99% purity) were sourced from Sigma-Aldrich (Sigma-Aldrich, St. Louis, MO, USA). For the organ bath study, all compounds were mixed and dissolved in distilled water. Fresh solutions were produced for each experiment.

### 4.2. Allergens

Allergens employed in the sensitization procedure include ovalbumin (grade V, Sigma-Aldrich, St. Louis, MO, USA), extracts of purified house dust mite (*Dermatophagoides pteronyssinus*, HDMs), and ragweed pollen (*Ambrosia artemisiifolia*, RW), both purchased from Allergon AB (Allergon AB, Thermo Fisher Scientific Inc., Ängelholm, Sweden). As adjuvant, aluminum hydroxide was utilized (1:100, *v*:*v* as allergen/Al(OH)_3_).

The house dust mite extracts were obtained from purified *Dermatophagoides pteronyssinus* mites, and an aqueous solution was prepared by dissolving 0.3 g of HDM extract in 5 mL of PBS, to which a protease inhibitor cocktail (Sigma-Aldrich, St. Louis, MO, USA) was added as per the provided guidelines. This solution was then homogenized using an ultraturax at 500× *g* force for 20 min at 4 °C on a heat block and then the mixture was stirred at 5 rpm overnight at 4 °C. The following day, the solution underwent centrifugation at 18,000× *g* force for 20 min at 4 °C. The final steps included dialysis with PBS and filtration through a 0.22 micrometer filter (Millex Millipore, Merck KGaA, Darmstadt, Germany). In a similar manner, the aqueous solution of purified *Ambrosia artemisiifolia* pollen was prepared in order to obtain the ragweed extract, in accordance with the protocol established by Buzan et al. and Grijincu et al. [26,58]. Aliquots from both extracts were stored then at −80 °C and utilized as needed.

The allergen doses were determined from an analysis of prior research, which demonstrated successful induction and expression of allergic asthma inflammation. All experiments were completed using identical batch of allergens.

### 4.3. Animals

Sixty male and female *Sprague Dawley* rats, with an average weight of 311.33 ± 15.26 g and an age range of 10–12 weeks, were procured from INCDMI “Cantacuzino” (Bucharest, Romania). The rats were housed in cages within a specially maintained room with a controlled temperature (22 ± 2 °C) and humidity (50% ± 5%), following a 12/12 h light/dark cycle (lights on between 08:00 and 20:00). They had ad libitum access to food and fresh water.

**Figure 7:**
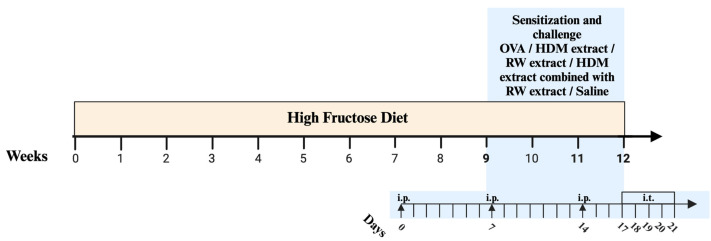
Experimental protocol. Sensitization and challenge protocol with OVA/HDM extract/RW pollen extract/HDM extract combined with RW pollen extract/saline. OVA = ovalbumin, RW = ragweed, HDM = house dust mite, i.p. = intraperitoneal injections; i.t. = intratracheal instillations.

The experimental cohort was randomly divided into ten groups, each consisting of six rats with an equal distribution of males and females. Among these, five groups were provided with a standard rat chow (the specifications are described in Table A1). The remaining five groups were subjected to a high-fructose diet, attained by having a standard diet combined with water fortified with fructose (a freshly prepared solution of 40% concentration, containing D-(−)-fructose and drinking water) that was changed every alternate day, following the protocol outlined by Pérez-Corredor et al. [29,33]. The induction of obesity with the forementioned high-fructose diet was maintained for a duration of 12 weeks. Eight of these groups were asthmatic and sensitized to at least one of the following allergens as described in Figure 7: ovalbumin, ragweed pollen extract, and house dust mite extract. The experimental protocol and each group’s allocation is shown in Figure 7.

Body weight was assessed at the beginning of the study and after 6 and 12 weeks using a digital scale. Measurement of abdominal circumference involved positioning the animal in a supine position with the legs slightly apart from the body and the measurement at the midsection of the abdomen.

Approval for animal experimentation was obtained from the Ethics Committee of the “Victor Babes” University of Medicine and Pharmacy in Timisoara (approval no. 41/20 December 2023), as well as from the King Michael I University of Life Sciences from Timisoara, Romania (approval no. 277/17 November 2023).

### 4.4. Quantification of Metabolic Parameters and Serum Inflammation Markers

Blood was collected from the rat’s lateral tail vein before the experiment and at the end of 12 weeks, before the scarification. It was then centrifuged at 3500 revolutions per minute (rpm) for 5 min at 4 °C. Serum was collected using pasture pipettes and stored at 4 °C until used in the same day in assays. Total cholesterol, serum triglycerides, total lipids, serum glycemia, C reactive protein, and total IgE levels were determined by serum spectrophotometry (with Beckman Coulter DxC 700 AU, Beckman Coulter Inc., Brea, CA, USA) using commercial kits in accordance with the manufacturer’s instructions.

### 4.5. Sensitization and Challenge Protocol

In order to induce allergic asthma, rats were sensitized either to ovalbumin, house dust mite, ragweed pollen, or to the combination of HDM and RW pollen extracts. Before each sensitization, a fresh suspension was prepared containing 100 μg allergen adsorbed on aluminum hydroxide (*v*:*v*, 1:100), diluted in sterile saline (0.9% NaCl). The suspension was homogenized gently in a rotator at 4 °C for 2 h so that the allergen could adsorb onto the Al(OH)_3_ [59]. On days 0, 7, and 14, the rats received intraperitoneal (i.p.) injections containing either 100 μg ovalbumin, 100 μg HDM extract, 100 μg RW extract, 50 μg HDM extract combined with 50 μg RW extract, or only saline for the unsensitized rats.

The allergen challenge was made for a period of 5 days consecutively, beginning on the 17th day. Prior to intratracheal allergen administration, the anesthesia was performed with 5% isoflurane mixed with oxygen followed by a 2% maintenance dose [60]. Animals were subjected to intratracheal instillations with 20 μg allergen in saline. For the combination of allergens, the rats received equally divided portions of HDM and RW extracts in the same quantity as mentioned above. At 24h after the final intratracheal challenge, the rats were euthanized, and subsequent examinations were conducted. The timing and concentrations of allergens were based mainly upon a review of previous publications demonstrating successful sensitization and induction of allergic inflammation in the lungs [2,3,28,50,59,61,62,63,64,65]. No visible adverse effects related to allergen administration were observed in the animals. The control group received i.p. injections and i.t. instillations with only saline solution

### 4.6. Tracheal Ring Preparation and Its Responsiveness Assessment

The animals were euthanized under general anesthesia through cervical dislocation. The trachea and lungs were directly dissected, extracted, washed, and cleaned by removing the surrounding connective tissue, then placed in cold buffer solution [66]. Every trachea was divided into two segments (with the length of 4–5 mm each) that were analyzed into the organ bath system.

**Figure 8:**
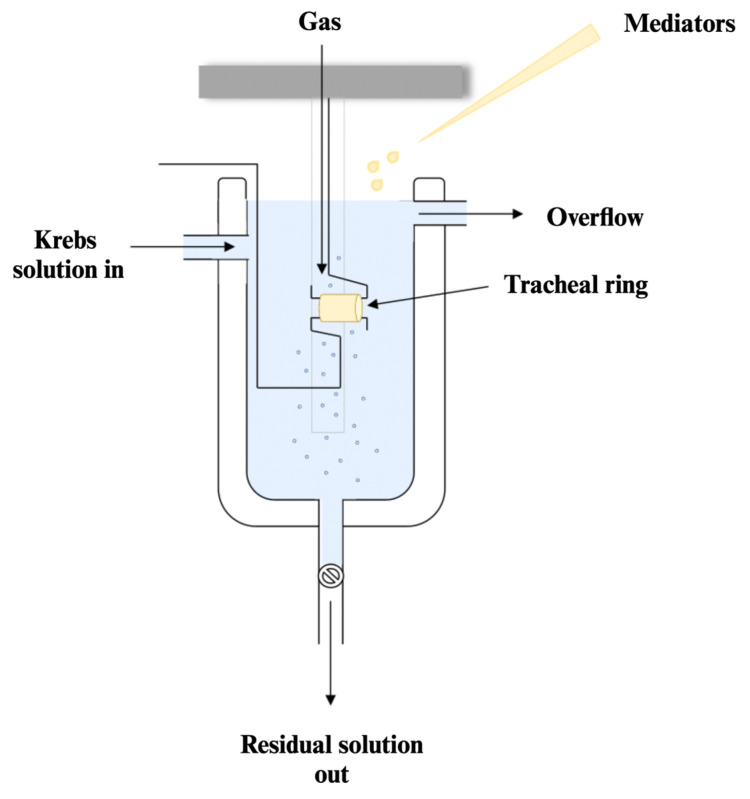
Representation of an organ bath chamber for tracheal rings.

Rat tracheal rings were mounted in the organ bath system (Figure 8), where the glass chambers were filled with 10 mL Krebs–Henseleit solution (its composition in mM: glucose 11; NaCl 118; KCl 4.70; KH_2_PO_4_ 2.15; MgSO_4_ 0.6; NaHCO_3_ 25; CaCl_2_ 1.69), heated at 37 °C, maintained at a pH of 7.4, and aerated with carbogen (a mixture of 95% O_2_ and 5% CO_2_) [67]. The four-channel force transducer (EXP-SG-4, Serial No: 1300363, Experimetria Ltd., Budapest, Hungary) was connected through the amplifier (SOFT-08-32 A1121015, Serial NO: 1300370, Experimetria Ltd., Budapest, Hungary) to the integrated tissue bath system (ISO-08, Serial No: 1300360, Experimetria Ltd., Budapest, Hungary) and displayed using the SPEL Advanced IsoSys v3.97 software (Experimetria Ltd., Budapest, Hungary). The ideal conditions needed in the organ chambers were strictly controlled with the aid of the heater circulating pump (Radnoti 170051G, ADInstruments Inc., Colorado Springs, CO, USA) and the carbogen gas cylinder (Linde Gaz, Timisoara, Romania).

Eventually, we measured the isometric tension generated by the tracheal rings and their responsiveness to the bronchoconstrictor drugs. Our preliminary trials and a survey of previous publications indicated that the optimal basal tension should be at 1.5 g force. After one hour of equilibration, the tissue viability and reactivity were tested twice with potassium chloride (KCl, 90 mM). The differences between smooth muscle hyperreactivity in each group were assessed by determining the maximum contractions to methacholine chloride. Increasing concentrations of mediator that ranged from 10^−8^ to 10^−4^ M were introduced into the organ bath every 2 min and the resulting contraction for each dose was then measured to generate a cumulative concentration–response curve. The tracheal muscle reactivity is determined as the value obtained by subtracting the basal tension measured before mediator administration from the maximum contraction recorded. Subsequently, the contraction response for each group and concentration was converted into a percentage relative to the mean contraction obtained in the control group at the 10^−6^ M concentration (997 milligram contraction force).

### 4.7. Histological and Morphometric Analysis of the Airways

The trachea and subsequent bronchopulmonary apparatus were retrieved after removing the necessary fragments for the organ bath experiment. Following fixation in 10% formaldehyde solution, the tissues underwent paraffin embedding and were sectioned to a thickness of 4–5 μm using a microtome. Cross-sections were performed for the tracheas and both lungs, which were stained with hematoxylin and eosin (H&E). Subsequent cross-sectional analyses of the trachea and lungs were conducted in a single-blind manner by a pathologist. Under 20× magnification, the stained airways were selected randomly and photographed using the Invitrogen EVOS^TM^ FL Auto 2 Imaging System (Thermo Fisher Scientific Inc., Bothell, WA, USA).

### 4.8. Statistical Analysis

All data were analyzed and are presented as the mean ± SD. The effect of each allergen sensitization, the diet, and their interactions on the methacholine contractions were analyzed by unpaired Student’s *t*-test or repeated measures two-way analysis of variance test (RM-ANOVA) as deemed appropriate, followed by Tukey and Bonferroni’s multiple comparison test. The statistical program used was GraphPad Prism version 8.3.1 (GraphPad Software, Boston, MA, USA). Statistical analysis for blood and anthropometric parameters was performed using one-way or two-way analysis of variance (ANOVA) followed by Tukey’s or Sidak’s multiple comparison test. In all experiments, “n” corresponds to the number of animals from which tracheal segments were extracted (6 in each instance). Statistical significance was defined as *p* < 0.05.

## 5. Conclusions

Our results demonstrate that a high-fructose diet adversely impacts allergic asthma in rats by inducing obesity, particularly abdominal obesity, and by aggravating local and systemic inflammation, leading to a more severe manifestation of the disease. Co-sensitization to HDMs and RW pollen resulted in the highest tracheal hyperresponsiveness in vitro and further worsened dyslipidemia and obesity features in both the HR and OHR groups. We also demonstrated a significant association between serum total IgE and serum CRP values in the context of allergic asthma and HFrD. This research highlights significant connections between allergic asthma and HFrD-induced obesity, emphasizing their combined impact through inflammation.

## Figures and Tables

**Table 1:** Weight evolution at 0, 6, and 12 weeks from the initiation of group-specific conditions. Results from each point are expressed as the mean value ± SD. The groups are depicted as follows: C = control; O = high-fructose diet; V = ovalbumin sensitization; OV = high-fructose diet and ovalbumin sensitization; R = ragweed pollen sensitization; OR = high-fructose diet and RW sensitization; H = house dust mite sensitization; OH = high-fructose diet and HDM sensitization; HR = HDM and RW sensitization; OHR = high-fructose diet and HDM and RW sensitization. Statistical analysis was performed using two-way analysis of variance (ANOVA) followed by Sidak’s multiple comparison test. *** *p* < 0.001 and **** *p* < 0.0001 vs. 0 weeks in each row.

Group	0 Weeks	6 Weeks	12 Weeks
C	309.50 ± 16.80	325.00 ±14.27	343.17 ± 14.92
O	311.83 ±11.82	362.17 ± 17.12 ****	405.83 ± 13.96 ****
V	310.17 ± 17.27	332.33 ± 14.19	331.33 ± 15.91
OV	306.83 ± 12.73	354.00 ± 15.82 ****	378.83 ± 17.09 ****
R	315.83 ± 18.27	330.83 ± 19.25	326.33 ± 20.76
OR	312.67 ±15.91	354.33 ± 14.75 ***	393.17 ± 12.32 ****
H	306.50 ± 15.73	319.67 ± 17.5	323.17 ± 19.73
OH	311.67 ± 18.68	359.67 ± 13.88 ****	380.83 ± 20.46 ****
HR	319.50 ±12.79	337.33 ± 16.16	310.17 ± 12.40
OHR	308.83 ± 18.59	350.67 ± 17.19 ***	363.17 ± 14.20 ****

## Data Availability

The original contributions presented in the study are included in the article, further inquiries can be directed to the corresponding author.

## Appendix A

**Table A1:** Description of standard rat chow according to manufacturer specifications—INCDMI “Cantacuzino” (Bucharest, Romania).

Category	Nutrient	UM	Quantity
General	Digestible energy	kJ/g	16.0
	Fat	g/kg	50.0
	Crude protein	g/kg	180.0
	Arginine	g/kg	4.3
	Asparagine	g/kg	4.0
	Glutamic acid	g/kg	40.0
	Histidine	g/kg	2.8
	Isoleucine	g/kg	6.2
	Leucine	g/kg	10.7
	Lysine	g/kg	9.2
	Methionine + Cystine	g/kg	9.8
	Phenylalanine + Tyrosine	g/kg	10.2
	Threonine	g/kg	6.2
	Tryptophan	g/kg	2.0
	Valine	g/kg	7.4
Minerals	Calcium	g/kg	5.0
	Magnesium	g/kg	0.5
	Chlorine	g/kg	0.5
	Phosphorus	g/kg	3.0
	Potassium	g/kg	3.6
	Sodium	g/kg	0.5
	Copper	g/kg	5.0
	Iodine	g/kg	0.15
	Iron	g/kg	35.0
	Selenium	g/kg	0.15
Vitamins	Thiamine (B1)	mg/kg	4.0
	Riboflavin (B2)	mg/kg	3.0
	Pyridoxine (B6)	mg/kg	6.0
	Cyanocobalamin (B12)	mg/kg	50.0
	Menadione (K)	mg/kg	1.0
	Retinol (A)	mg/kg	1.2
	Cholecalciferol (D3)	mg/kg	25.0
	Tocopherol (E)	mg/kg	27.0
	Choline	mg/kg	750.0
